# Co-delivery of genes can be confounded by bicistronic vector design

**DOI:** 10.1557/s43579-021-00128-7

**Published:** 2022-02-18

**Authors:** Hanieh Moradian, Manfred Gossen, Andreas Lendlein

**Affiliations:** 1 Institute of Active Polymers, Helmholtz-Zentrum Hereon, 14513 Teltow, Germany; 2 Berlin-Brandenburg Center for Regenerative Therapies (BCRT), 13353 Berlin, Germany; 3 Institute of Biochemistry and Biology, University of Potsdam, 14476 Potsdam, Germany

**Keywords:** Molecular, Packaging, Protein

## Abstract

Maximizing the efficiency of nanocarrier-mediated co-delivery of genes for co-expression in the same cell is critical for many applications. Strategies to maximize co-delivery of nucleic acids (NA) focused largely on carrier systems, with little attention towards payload composition itself. Here, we investigated the effects of different payload designs: co-delivery of two individual “monocistronic” NAs versus a single bicistronic NA comprising two genes separated by a 2A self-cleavage site. Unexpectedly, co-delivery via the monocistronic design resulted in a higher percentage of co-expressing cells, while predictive co-expression via the bicistronic design remained elusive. Our results will aid the application-dependent selection of the optimal methodology for co-delivery of genes.

**Graphical abstract:**

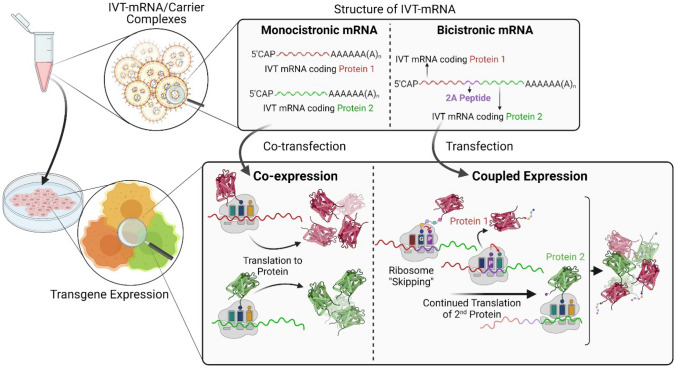

**Supplementary Information:**

The online version contains supplementary material available at 10.1557/s43579-021-00128-7.

## Introduction

Delivery of recombinant nucleic acids (NA) into cells is an essential process in gene therapy^[^^1^^]^ as well as genetic engineering for basic research.^[^^2^^]^ Robust delivery systems typically consist of polymeric or liposomal nanocarriers with physically bound RNA or DNA, including therapeutic NAs,^[^^3,4^^]^ but also vaccines,^[^^5^^]^ as highlighted by the response to the recent SARS-CoV-2 global outbreak.^[^^6^^]^ The NA payload depends on the requirements of the application, which in many cases demand expression of multiple transgenes. To rapidly identify expression via live cell imaging, target transgene can be coupled with a fluorescence reporter gene, also facilitating subsequent cell sorting.^[^^7,8^^]^ When production of large multisubunit proteins like antibodies is aimed, multiple genes coding for different subunits must be simultaneously delivered to the same cell.^[^^9^^]^ The same applies for the expression of enzyme complexes.^[^^10^^]^ Also (re)programming cells fate requires combinatorial expression of transcription factors.^[^^11–13^^]^ This includes the dedifferentiation of somatic cells into induced pluripotent stem cells (iPSCs),^[^^14^^]^ or the transdifferentiation to another somatic cell lineage.^[^^15^^]^ Genome editing by CRISPR/Cas9 technology even requires delivery of three NA types including a guide RNA (gRNA), a gene (mRNA/pDNA) coding Cas9 protein, and optionally the DNA donor for targeting are crucial to successfully perform.^[^^7,16^^]^

There are various strategies to achieve simultaneous delivery of multiple NAs, and thus co-synthesis of multiple proteins in the same cell, including (i) incorporation of multiple transcription units within the same vector,^[^^17,18^^]^ (ii) fusion of genes,^[^^19^^]^ (iii) introduction of internal translation initiation sites such as internal ribosome entry sites (IRES),^[^^20^^]^ (iv) inserting enzyme-dependent cleavage sites in polyproteins,^[^^21^^]^ and (v) enzyme-independent (apparent) self-cleavage sites between genes.^[^^22,23^^]^ The first method is only applicable for gene delivery via plasmid DNA (pDNA), but not messenger RNA (mRNA). Moreover, the introduction of post-translational enzymatic cleavage sites is restricted to co-localization of enzyme and protein. Therefore, cap-independent internal initiation sites, e.g. IRES, and enzyme-independent (apparent) self-cleavage peptides such as 2A peptides gained considerable popularity, both result in multi-gene expression within a single cassette, also referred to as “multicistronic” genes. When encountering 2A sequences, ribosomes skip the formation of the peptide bond between glycine and proline amino acids of the 2A peptide, only to continue with translation of the second gene. Thus, in theory, this vector design should inherently result in a 1:1 molar stoichiometry of the two nascent polypeptide chains, while the equilibrium of the proteins in question might diverge. In any case, connecting genes on the NA level by 2A peptides encoding sequences should guarantee the co-synthesis of either of the proteins in a transfected cell, and was reported to result in reliable co-expression when evaluated empirically.^[^^24^^]^ Of note, while the multicistronic approach ensures equimolar representation of the genes in question, the effective intracellular concentration of the encoded proteins may differ substantially, especially due to differences in protein stability. Alternatively, two distinct monocistronic genes could be packaged within the same carrier, and being taken up and co-expressed by the same cell.^[^^25,26^^]^

**Figure 1:**
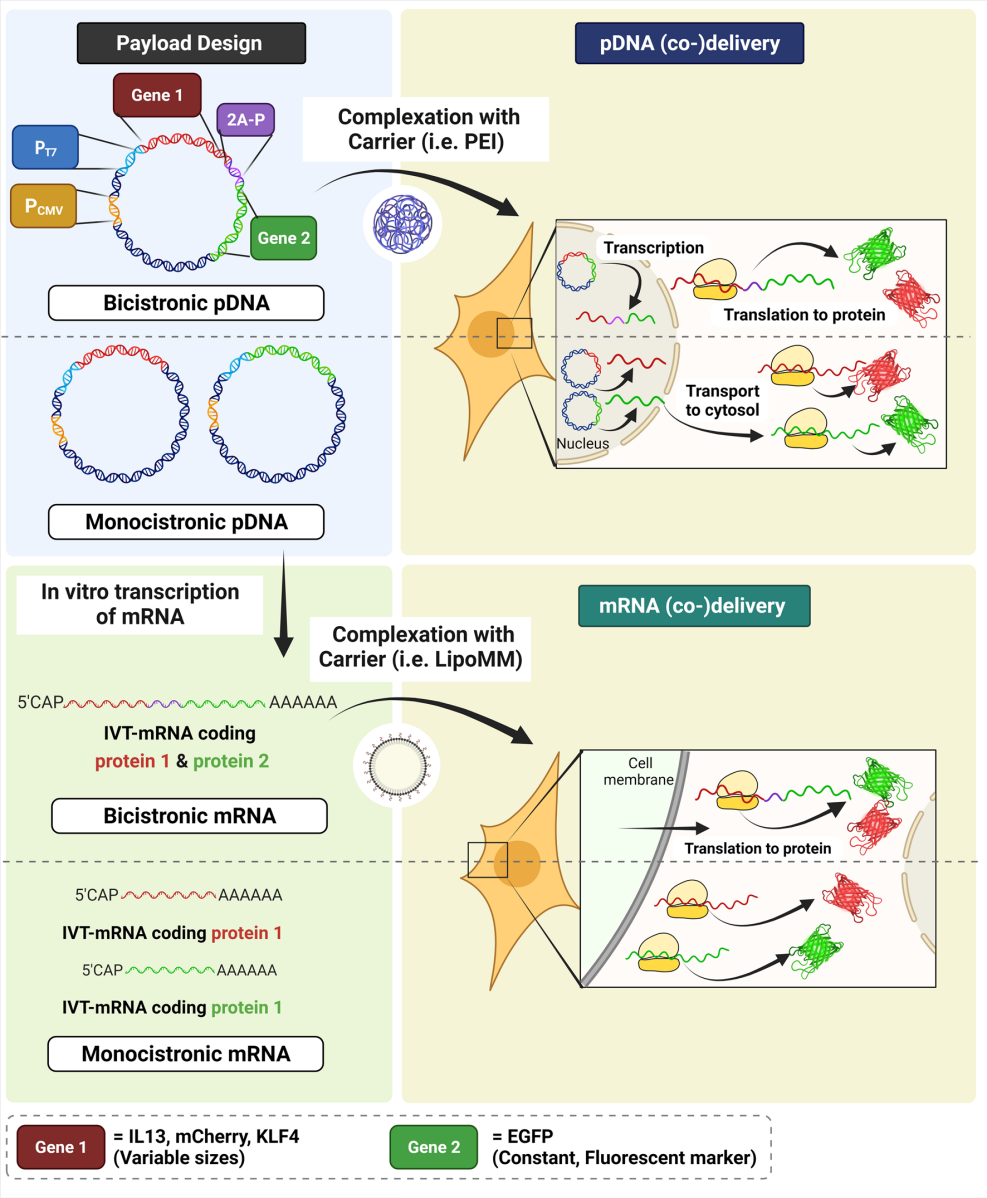
Schematic representation of the study design to achieve nucleic acid-directed co- production of target proteins in individual cells. Plasmid DNA was directly (co-)delivered to cells using an established carrier system (i.e. PEI) (upper panel) or used as a template to IVT-mRNA, followed by (co-)delivery of IVT-mRNA by LipoMM to cells (lower panel). The former requires nucleus entry and transcription to mRNA, whereas in the latter case IVT-mRNA is, upon cellular uptake and endosomal escape, instantly translated to protein in the cytoplasm (not illustrated here). In both cases, however, mRNA is the ultimate entity, which is processed by ribosomes as a blueprint for protein synthesis. The two distinct payload designs, namely monocistronic and bicistronic nucleic acid refer to two genes integrated within one continuous open reading frame, and different IVT-mRNAs co-formulated together in a single carrier type (e.g., lipoplex or polyplex) in a statistical fashion, respectively. Three different genes with distinct sizes were assessed as the first gene, while keeping EGFP as a fluorescent marker constantly as the second gene. The order of representation does not reflect the experimental sequence. (Illustration created by BioRender.com).

The aim of this study was to find the most reliable and robust gene co-delivery approach for simultaneous production of two proteins in the same cell, by comparing two commonly used strategies including delivery of a “bicistronic” gene versus co-delivery of two distinct “monocistronic” genes. We hypothesized that co-expression of two transgenes directly coupled by 2A-design should be most efficient to ensure predictable synthesis of both the corresponding proteins in a cell, due to the inherently equivalent molar ratio of the two genes encoded in the same open reading frame, as one transcription unit with continuous ribosomal protein synthesis. This notion was empirically investigated by systematic side-by-side comparison of cells either transfected with nucleic acid comprising of two genes separated by a 2A peptide, or co-transfected with two separate nucleic acids as a control (Fig. 1). These experiments were initially performed by direct comparison of equimass versus equimolar ratio of the two genes, as only possible and reasonable for the monocistronic approach and evaluated by transfection of in vitro transcribed mRNA (IVT-mRNA) as payload, followed up by equimass ratio analysis only. The latter was also investigated by implementing pDNA as genetic payload for a selected set of experiments. Given that the initial quantitative co-expression via the 2A approach was confounded, we further evaluated the effect of the size of the first gene on co-expression rates and the potential cell-type specificity of this effect. Establishing effective methods for co-expression of multiple transgenes could be beneficial in addressing complex gene delivery studies.

## Materials and methods

### Design of pDNA vectors

pRNA2-(A)128 plasmid DNA vector comprising a CMV promoter, a T7 promoter, a short intron-less 5’-untranslated region (UTR) with Kozak sequence, enhanced green fluorescent protein (EGFP) coding region (or other open reading frames in case of pRNA2-derivatives used in this study), a head-to-tail duplicated human β-globin 3′-UTR providing increased transcript stability, followed by a homopolymeric 128-base polyadenine stretch (for use as a template in the IVT reaction and a poly(A) signal (for direct use of the plasmid in DNA transfections); all as described previously^[^^27^^]^ (Figure S1). Thus, identical pRNA2 constructs and their derivatives are dual-use for IVT-mRNA and pDNA applications. In order to induce simultaneous expression of two genes upon cellular (co-)delivery, DNA templates were designed according to two different approaches described as follows:

(i) ** Monocistronic genes** comprised of only one gene in a single cassette (Figure S1 left panel). Enhanced green fluorescent protein (EGFP) coding region in pRNA2-(A)_128_ vector mentioned above was replaced with one of the following genes: red fluorescent protein (mCherry), interleukin 13 (IL13), or Krueppel-like factor 4 (KLF4), to generate pRNA2-(A)_128_-mCherry, pRNA2-(A)_128_-IL13, or pRNA2-(A)_128_-KLF4, respectively.
(ii) **Bicistronic genes** consist of two genes in the same cassette separated by a 2A peptide sequence (Fig. 1, upper panel, Fig. S1, right panel). Among different existing 2A peptide sequences, the P2A peptide was selected in this study, due to superior performance as reported previously.^[^^28^^]^ Consequently, the three above-mentioned genes, i.e. mCherry, IL13 and KLF4 coding genes, were coupled with EGFP expressing sequence in a single vector, separated by a P2A peptide containing amino acid sequence of GSGATNFSLLKQAGDVEENPGPKL. The resulting vectors were referred to as pRNA2-(A)_128_-mCherry-2A-EGFP, pRNA2-(A)_128_-IL13-2A-EGFP, and pRNA2-(A)_128_-KLF4-2A-EGFP, respectively.

### mRNA synthesis by in vitro transcription

Monocistronic and bicistronic mRNAs were individually synthesized by in vitro transcription using the above-mentioned pDNAs. mRNAs were synthesized according to our previously published protocol.^[^^29^^]^ Briefly, plasmid vectors were linearized with BspMI restriction enzyme (New England Biolabs, Germany), and precipitated using a salt mixture including 0.05 Vol 3 M sodium acetate (Thermo Fisher Scientific, Germany), in presence of 0.1 Vol 0.5 M ethylenediaminetetraacetic acid (EDTA) (Thermo Fisher Scientific), and 2 Vol 100% EtOH (Carl Roth, Karlsruhe, Germany). Subsequently, mRNAs were synthesized using TranscriptAid T7 High Yield Transcription Kit (Thermo Fisher Scientific) according to the manufacturer’s instruction. Of note, the 5′ end of IVT-mRNA was co-transcriptionally modified with anti–reverse cap analog (ARCA) (Jena Bioscience, Germany). For transfection of macrophages, chemically modified IVT-mRNAs coding for mCherry, EGFP and mCherry-2A-EGFP were prepared by complete substitution of uridine and cytidine with pseudouridine (Jena Bioscience) and 5-methylcytidine (Jena Bioscience), respectively. IVT-mRNAs were purified using lithium chloride precipitation and resuspended in UltraPure™ nuclease-free sterile water (Merck Millipore, Germany) supplemented with 0.1 mM EDTA. The concentration of IVT-mRNA products was determined by UV/Vis-spectroscopy (NanoDrop 1000 Spectrophotometer; Peqlab, Germany). Moreover, the quality/integrity of transcripts were assessed by denaturing agarose gel electrophoresis.

### Transfection of IVT-mRNA

Lipofectamine MessengerMAX (LipoMM; Thermo Fisher Scientific), a commercially available, lipoplex-forming reagent optimized for RNA transfections, was selected as a reagent for delivery of IVT-mRNAs. The IVT-mRNA/LipoMM complexes, required for co-delivery/co-transfection of monocistronic genes (MonoCis (CoTF)) or delivery of a bicistronic 2A peptide-comprising gene (BiCis (2A-P)), were prepared as follows; MessengerMAX reagent was diluted 1:50 (vol) in 125 µL Opti-MEM reduced serum medium (Thermo Fisher Scientific), and incubated for 10 min at RT. The resulting solution was added to the equal volume of Opti-mem containing defined amount of IVT-mRNA, individually or in mixture in case of co-transfection; see Table S1 for the precise amounts of IVT-mRNA used for equimass versus equimolar experiment when following the monocistronic approach. Briefly, for equal mass transfections either 500 ng of the respective BiCis IVT-mRNA or 250 ng of each of the 2 MonoCis IVT-mRNAs, as indicated, were used. For equal mole transfections the number of molecules for each of the MonoCis IVT-mRNA was equivalent to the molar amounts of 500 ng of the respective BiCis mRNA (Table S1). The monocistronic EGFP encoding IVT-mRNA (length: 1253 nts; i.e. 500 ng/well equals 1.24 pmol/well of 12-well plate) was used as a reference point throughout. For the transfection of cells in 6-well plates in case of equimass experiments, 1 µg IVT-mRNA, i. e. 1 µg bicistronic IVT-mRNA, or 500 ng from each of the two different IVT-mRNAs (MonoCis) premixed together (equal mass approach), or 1 µg combined total of the KLF4 and EGFP IVT-mRNA in the indicated ratios for the equal mole approach was used to prepare BiCis (2A-P), and MonoCis (CoTF) complexes, respectively. The mixture was briefly vortexed and incubated for 5 min at RT. Subsequently, the transfection complexes containing 1 µg IVT-mRNA in overall 250 µL Optimem were added to each well of HeLa (ATCC; CCl-2) cells in a 6-well plate format. HeLa cells were pre-seeded at a density of 3.00E + 05 cells per well of 6-well plates, in high glucose DMEM, supplemented with GlutaMAX™, pyruvate (Gibco, Germany), 10 vol% FBS (Biochrom, Germany) and 1 U∙mL^−1^ Penicillin–Streptomycin (Gibco, Germany), 24 h before transfection with IVT-mRNA.

### Quantitative analysis of cells with flow cytometry

Cells were harvested at above specified time points with TrypLE Select (Thermo Fisher Scientific). Upon washing with cold flow cytometry washing solution (Miltenyi Biotec, Germany), cells were analyzed with a MACSQuant VYB® flow cytometer (Miltenyi Biotec, Germany).

Production of non-fluorescent protein, e.g. KLF4, was detected by immunocytochemistry, using eBioscience ™ Foxp3 /transcription factor staining buffer set (Thermo Fisher Scientific), according to manufacturer’s instruction. The KLF4 protein was subsequently stained with recombinant Alexa Fluor® 647 anti-KLF4 antibody (Abcam, Germany). Cells were measured by MACSQuant VYB® flow cytometer. All flow cytometric data were analyzed with FlowJo software V10.

### Statistics

Data are presented as means ± standard deviation (SD) of at least three independent experiments. In case of primary human macrophages, three independent experiments were performed using cells derived from three different donors. Data were statistically analyzed via Prism 7.00 software (GraphPad, USA).

## Results and discussion

### Study design

The reliability of two methods differing in payload design was investigated in a series of side-by-side experiments. Two separate monocistronic genes were co-transfected in the first approach, whereas a single bicistronic gene coding both proteins in a single cassette, separated by a 2A peptide was delivered in the second approach. Herein, the former is referred to as “MonoCis (CoTF)”, while the latter is named “BiCis (2A-P)” throughout all experiments. As we previously validated the reliability of MonoCis (CoTF) approach,^[^^26^^]^ this condition was mainly included as reference and control to determine the performance of BiCis (2A-P) method. Throughout this study a fluorescent marker protein, i.e. enhanced green fluorescent protein (EGFP) was selected as the second protein, in order to enable facile and prompt monitoring of gene expression, investigated by fluorescent microscopy and quantified via flow cytometry. Experiments were designed to evaluate the effect of several parameters including cell type, NA identity (RNA vs. DNA) and size of the first gene on co-expression rate. The molecular weights of nucleic acids and also the respective genes implemented in this study are summarized in Table I. Addressing the latter parameter was spurred by our initial, unexpected observation, where inconsistent patterns of co-expression were monitored at single cell resolution for the BiCis (2A-P) approach.

**Table I:** Characteristics of the genes investigated in this study.

Gene Name	Description/function	Molecular Weight of pDNA vector (kDa)	Molecular Weight of IVT-mRNA (kDa)	Molecular weight of ORF^a^ pDNA (kDa)	Molecular weight of ORF^a^ mRNA (kDa)
EGFP	MonoCis/fluorescent marker	3201	401	444	231
IL13	MonoCis/control for small size gene	2979	286	271	141
mCherry	MonoCis/fluorescent marker/control for medium size gene	3165	382	439	228
KLF4	MonoCis/control for large size gene	3590	603	873	453
IL13-2A-EGFP	BiCis/co-expression of a gene with marker	3467	539	760	394
mCherry-2A-EGFP	BiCis/co-expression of a gene with marker	3634	625	927	481
KLF4-2A-EGFP	BiCis/co-expression of a gene with marker	4077	855	1370	711

^a^*ORF* open reading frame.

### Size of first gene does not correlate with co-expression rate

**Figure 2:**
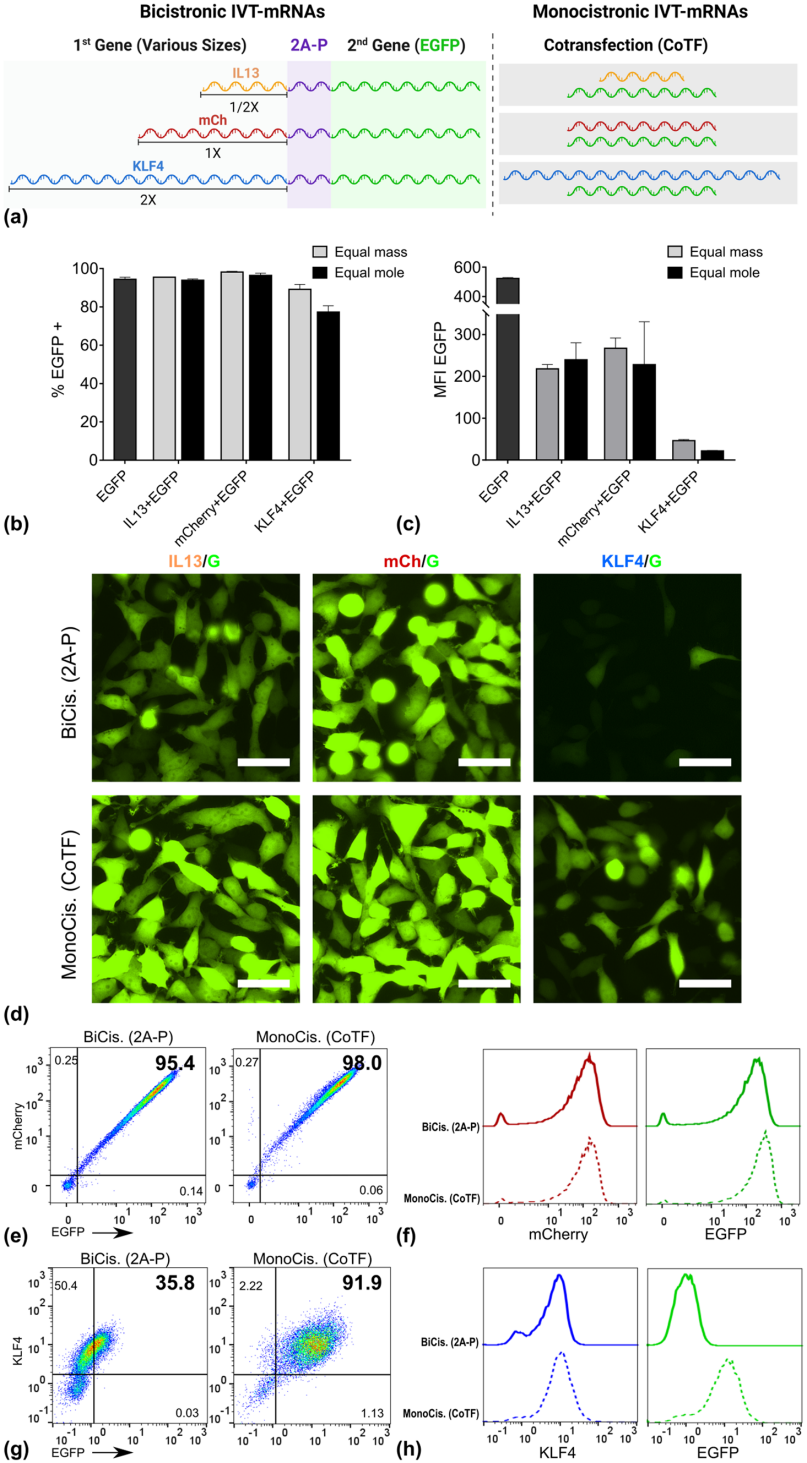
Impact of size ratio of the first gene to the second gene on the co-expression of two genes. (a) schematic overview of constructs, which were used and compared in parallel, IL13 with half size of EGFP as the small, mCherry with similar size to EGFP as medium, and KLF4 with almost double size of EGFP as large constructs were delivered together with EGFP either in one cassette with 2A-P as self-cleavage site, or co-delivered. For the monocistronic approach the percentage of EGFP positive population (b) and intensity of EGFP signal (mean fluorescent intensity) (c) reflecting the EGFP expression were quantified using flow cytometric evaluation of cells. Corresponding data for the bicistronic approach are provided in Figure S2. (d) Fluorescent images of HeLa cells transfected with any of the three genes with different sizes with two different approaches. Percent of double positive cells shown in form of dot plots (e), as well as expression level of the first protein (mCherry) and second protein (EGFP) compared between the two methods (f). Similarly, percent of double positive cells (g) and evaluation of protein production for KLF4 as first protein and EGFP as second protein and (h), both measured via immunocytochemistry (ICC) and flow cytometry. Numbers indicated within dot plots represent % of cells inside the corresponding gate. Error bars indicate SD for three independently performed experiments. Scale bar = 50 µm. See Table S1 for precise numbers describing equimass versus equimolar transfection, corresponding to each method.

As one possible explanation we investigated the effect of first gene’s size on co-expression rate of two genes, as well as expression level of the second gene. Three different IVT-mRNAs coding for IL13, mCherry (mCh), and KLF4 were selected, exemplifying the small (1/2 X), medium (1 X), and large (2 X) sizes, respectively, when compared to the size of the second gene, i.e. EGFP (G) (Table I, Fig. 2(a)). Note that for IL13 and KLF4 the choice of these genes was solely based on their size, not a potential biological function. These genes were either placed in position 1 upstream of EGFP in a single cassette, represented as BiCis (2A-P) (Fig. 2(a), left panel), or co-packaged as separate units each with EGFP within the same complex, named MonoCis (CoTF) (Fig. 2(a), right panel).

Given an identical mass of different IVT-mRNAs, those with smaller size correspond to larger numbers of molecules compared to that of larger size. Thus, a series of preliminary experiments with equal mass as well as equal moles were performed, to identify the reliable experimental set-up, and to avoid any potential misinterpretation of EGFP expression. The percent of EGFP positive cells and intensity of EGFP signal were measured by flow cytometry in side-by-side experiments, and presented for each condition (Fig. 2(b), (c)). The results demonstrated that the observed reduction of co-expression rate for a large gene in position 1 is not caused by its underrepresentation in terms of molar concentration when following an equal mass co-transfection protocol (Fig. 2b). Thus, for the subsequent experiments, the equal mass method was implemented throughout. The corresponding molar amounts for each condition is presented in Table S2.

The expression level of EGFP in HeLa cells transfected with either of these approaches was evaluated by fluorescent microscopy (Fig. 2(d)). No consistent pattern of variation in EGFP intensity was observed with respect to the size of the first gene, in cells transfected with BiCis (2A-P) IVT-mRNAs. While mCh/G resulted in higher EGFP expression than IL13/G, extremely only a strongly diminished signal was detected for KLF4/G (Fig. 2d, upper panel). However, MonoCis (CoTF) consistently led to a higher EGFP expression when compared to the BiCis (2A-P) approach (Fig. 2d, lower panel).

The co-expression rate as well as expression of the first protein were evaluated by flow cytometry (Fig. 2e-h). Data suggested no differences in percent of double positive cell population between cells transfected with mCh/G via two distinct methods. Unexpectedly, however, MonoCis (CoTF) was superior to BiCis (2A-P) in cells transfected with KLF4/G in terms of co-expression rate (Fig. 2g). When analyzed individually, there were no differences in production level of the first protein, namely mCh and KLF4 in mCh/G and KLF4/G transfected cells, respectively, between BiCis (2A-P) and MonoCis (CoTF) methods (Fig. 2f & h, left panel). However, as mentioned above, level of second protein production in the BiCis (2A-P) approach was remarkably lower compared to the MonoCis (CoTF) for KLF4/G (Fig. 2h, right panel).

**Figure 3:**
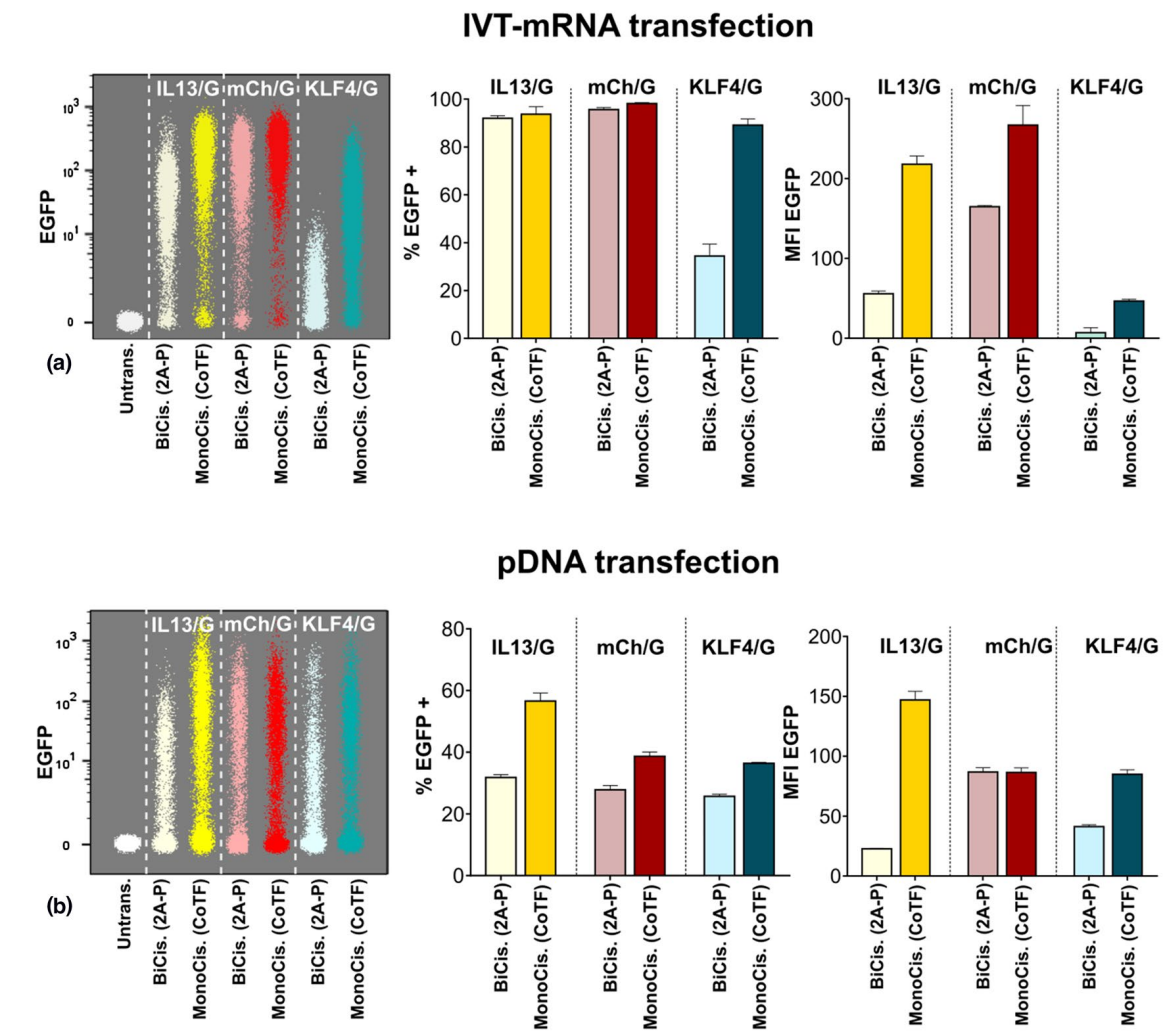
Quantification of transgene expression in BiCis versus MonoCis configuration. (a) Flow cytometric analysis of HeLa cells transfected with IVT-mRNA via the two methods; Left panel: Merged dot plot of cells expressing EGFP evaluated by flow cytometry. Middle panel: The percentage of EGFP positive cells. Right panel: The level of EGFP expression quantified from flow cytometry data. (b) Side-by-side comparison of EGFP expression in HeLa cells transfected by transgene-encoding plasmid DNA; Left panel: Merged dot plot of different genes expressed in HeLa cells measured by flow cytometry. Middle panel: Transfection efficiency indicated as percent of EGFP positive cells. Right panel: The level of transgene expression quantified by mean fluorescent intensity of EGFP signal. Cells were analyzed 24 h after IVT-mRNA transfection, and 48 h after pDNA transfection. Values are presented as mean ± SD, *n* = 3. Error bars indicate SD.

The EGFP expression quantified with flow cytometry (Fig. 3a), both in terms of percent of EGFP positive cells (Fig. 3a, middle panel), and intensity of EGFP signal representing level of expression within population of EGFP positive cells (Fig. 3a, right panel) was remarkably lower in BiCis (2A-P) compared to MonoCis (CoTF) for all three sizes. In particular, the minimum level of expression corresponded to KLF4/G both for MonoCis (CoTF), and more dramatically for BiCis (2A-P) methods (Fig. 3a, left panel).

Since the IVT-mRNA coding for KLF4 is almost twice as large as EGFP coding IVT-mRNA, the co-transfection with the MonoCis approach was also evaluated with ratios other than 1:1 (1:1.5), specifically 9:1 (6:1) and 1:9 (1:13.6), referring to KLF4: EGFP IVT-mRNA mass ratio (values in parenthesis referring to the molar ratio). Expression of both proteins were subsequently measured and plotted for each condition (Figure S3). Transfection of cells with MonoCis (CoTF) approach consistently led to higher percent of double positive cells, particularly remarkable also when the lowest ratio (i.e. 9:1) of EGFP was delivered (Figure S3).

These findings are consistent with previous report by Liu et al.^[^^12^^]^, where they also found puzzling patterns of second protein production, examined for different types of 2A peptides in multicistronic genes. While originally analyzing the efficacy of different 2A sequences, they also observed striking differences of EGFP expression in multicistronic reprogramming vectors, when placed in different positions relative to the other genes.^[^^12^^]^ The lower expression of second gene could potentially be attributed to detachment of ribosomes from messenger RNA, upon translation of first protein, as suggested by Shaimardanova et al.^[^^30^^]^. This was consistent with our observations, where no changes were detected in production of first protein, along with diminished production of second protein, particularly noticeable in KLF4/G transfected cells.

To investigate whether the differences observed between the two methods depend on the target cell-type, primary human monocyte-derived macrophages were transfected with mCh/G via both BiCis (2A-P) and MonoCis (CoTF) methods. Transfected macrophages were analyzed 24 h post-transfection for expression of the fluorescence marker proteins via fluorescent microscopy (Figure S4a), as well as flow cytometry (Figure S4b-f). There was no obvious difference between macrophages transfected with either method in terms of expression of mCherry and EGFP, as shown in individual channels (Figure S4a, left and middle panel), and merged fluorescent images (Figure S4a, right panel). However, when quantified with flow cytometry, the production level of both proteins was slightly lower in macrophages transfected with BiCis (2A-P) compared to MonoCis (CoTF) approach (Figure S4c, e, f). The co-expression rate, i.e. percent of double positive cells, were consistently lower for BiCis (2A-P) compared to MonoCis (CoTF) approach (Figure S4b, d). These findings argue against a cell-type dependence of co-expression patterns according to the approach chosen. However, the consistent differences between the two methods were interestingly more pronounced in primary cells than in established cell line, e.g. HeLa cells, which emphasized the importance of selection of proper payload design to achieve a reliable co-expression.

### Plasmid DNA transfection results in 2nd gene expression pattern similar to mRNA transfections

Next, we asked whether the differences observed between cells transfected with the two methods based on various payload design were exclusive to IVT-mRNA or could it be generalized to other nucleic acid entities, in particular plasmid DNA (pDNA) used in this study. Similar to IVT-mRNA, bicistronic pDNA was comprised of both genes at the same plasmid separated by 2A peptide, whereas monocistronic pDNA coding for each gene had to be co-delivered. The experiments were performed by using equimass transfection of pDNA (Table S2). The level of second protein production in HeLa cells was measured using flow cytometry as the key readout (Fig. 3b). Interestingly, no correlation was identified between size of first gene and the expression of the second gene coding EGFP, as indicated by side-by-side comparison of pDNA samples with increasing size of the first gene, i.e. IL3/G, mCh/G, and KLF4/G, respectively (Fig. 3b, left panel). Transfection efficiency determined as percent of EGFP positive cells (Fig. 3b, middle panel), and level of protein production quantified and defined as mean fluorescent intensity (MFI) of EGFP signal (Fig. 3b, right panel) were compared in cells transfected with pDNA. Data suggested that BiCis (2A-P) consistently resulted in lower transfection efficiency and protein production level of the second protein, when compared to MonoCis (CoTF) approach (Fig. 3b). Overall, comparison of data between pDNA transfection (Fig. 3b) with IVT-mRNA transfection (Fig. 3a) revealed that the differences observed between two methods is not dependent on the chemical identity of the chosen NA nor its biophysical properties, i.e. linear with relaxed topology for IVT-mRNA as compared to circular, supercoiled pDNA. However, the extent to which the expression differs is more pronounced for IVT-mRNA transfection; See left panels in Fig. 3a& b.

## Conclusions

We implemented two methods for simultaneous delivery of two distinct genes based on different payload compositions. A systematic comparison of a bicistronic gene coding for two proteins separated by a 2A peptide sequence side-by-side with the co-delivery of two separate genes revealed that the latter is the more reliable approach. While a bicistronic design will lead to a one-to-one stoichiometry of templates for protein synthesis independent of formulation and uptake route, experimental evidence on the level of proteins actually synthesized argued against this notion. Moreover, none of the envisaged and examined parameters including the first protein’s size, cell-type, or nucleic acid identity (IVT-mRNA vs. pDNA) had a determining role on co-expression rates. In contrast, co-delivery of two monocistronic genes consistently resulted in robust expression of the second protein, proved to be true throughout all tested conditions with different gene sizes, for both IVT-mRNA and pDNA, among different cell-types. As the underlying mechanisms contributing to the observed erratic performance of the 2A peptide remain to be solved, we can only speculate about potential effects of nucleic acid sequence and the corresponding secondary structure of mRNA around the 2A peptide, or the conformation of first nascent protein on tendency of ribosomes whether to continue translation of the second protein or to disengage from the mRNA and thus stop translation. In any case, it is obvious that the 2A sequence can affect protein-synthesizing ribosomes in a way that it impedes on their scheduled translation path.

## Supplementary Information

Below is the link to the electronic supplementary material.

Supplementary file1 (PDF 1036 kb).

## Data Availability

The datasets generated during and/or analysed during the current study are available from the corresponding author on reasonable request.
