# Supplementary material for: Co-delivery of genes can be confounded by bicistronic vector design
Source: MRS Commun. 2022 Feb 18;12(2):145–53. doi: 10.1557/s43579-021-00128-7 (PMC8856875; doi:10.1557/s43579-021-00128-7)
Supplement: Supplementary file 1 — Supplementary file1 (PDF 1036 kb). [file 43579_2021_128_MOESM1_ESM.pdf]

Co-delivery of genes can be confounded by bicistronic vector design

Hanieh Moradian[1, 2, 3], Manfred Gossen[1, 2], Andreas Lendlein[1, 2, 3,*]

1 Institute of Active Polymers, Helmholtz-Zentrum Hereon, 14513 Teltow, Germany
2 Berlin-Brandenburg Center for Regenerative Therapies (BCRT), 13353 Berlin, Germany
3 Institute of Biochemistry and Biology, University of Potsdam, 14476 Potsdam, Germany

* Corresponding Author, email address: lendlein@uni-potsdam.de

A) Supplementary Materials and Methods

Transfection of macrophages with IVT-mRNA

mCherry, EGFP and mCherry-2A-EGFP coding mRNAs were also examined in primary human macrophages. Macrophages were differentiated *in vitro* from monocytes purified from buffy coat-derived peripheral blood mononuclear cells (PBMCs) (Deutsche Rote Kreuz, Berlin; ethics vote EA2/018/16; Charité University Medicine Berlin) as previously described [1]. Macrophages were transfected with complexes containing chemically modified IVT-mRNAs. Respective volumes of the above prepared complexes were transferred to each well to deliver 125 ng/mL mRNA in cell culture medium. Macrophages were transfected at a density of 2.00E+06 cells per well of 6 well plates in 2 mL very low endotoxin (VLE) RPMI medium (Biochrom) supplemented with 10 vol% FBS.

Transfection of plasmid DNA (pDNA)

Being equipped with a CMV promoter, the pDNA templates were also directly transfected into HeLa cells. Plasmids were complexed and delivered via transfection-grade 25 kDa linear polyethylenimine (PEI) (Polyscience, Germany), a commercially available, polyplex-forming transfection reagent routinely used for delivery of plasmid DNAs, according to a previously published protocol [2]. Briefly, 2 µg pDNA diluted in 50 µL of 150 mM NaCl was combined with 7.5 mM PEI at nitrogen/phosphate (N/P) molar ratio of 20. pDNA solution was prepared either with 2 µg of single bicistronic pDNA or by pre-mixing 1 µg from each of two monocistronic pDNAs, referred to as BiCis (2A-P) and MonoCis (CoTF) transfection, respectively. The resulting mixture containing 2 µg pDNA was vortexed for 30 sec, and incubated for 10 min at RT for complex formation and then added dropwise to each well. HeLa cells were pre-seeded at a density of 2.50E+05 cells per well of 6-well plates 24 h before transfection.

Evaluation of cells with fluorescent microscopy

To evaluate fluorescent proteins production, cells were imaged with an inverted microscope ELIPSE Ti-U (Nikon) equipped with a mercury light source, Intensilight C-HGFI, and single-band filter sets; EGFP-BP filter (466/40, 525/50 nm), or TRITC filter (562/40, 641/75 nm) (Semrock, Germany). Images were acquired 24 and 48 h after cells transfection with IVT-mRNA or pDNA, respectively. All images were analyzed with NIS-Elements imaging software, version 4.51 (Nikon).

B) Supplementary Data

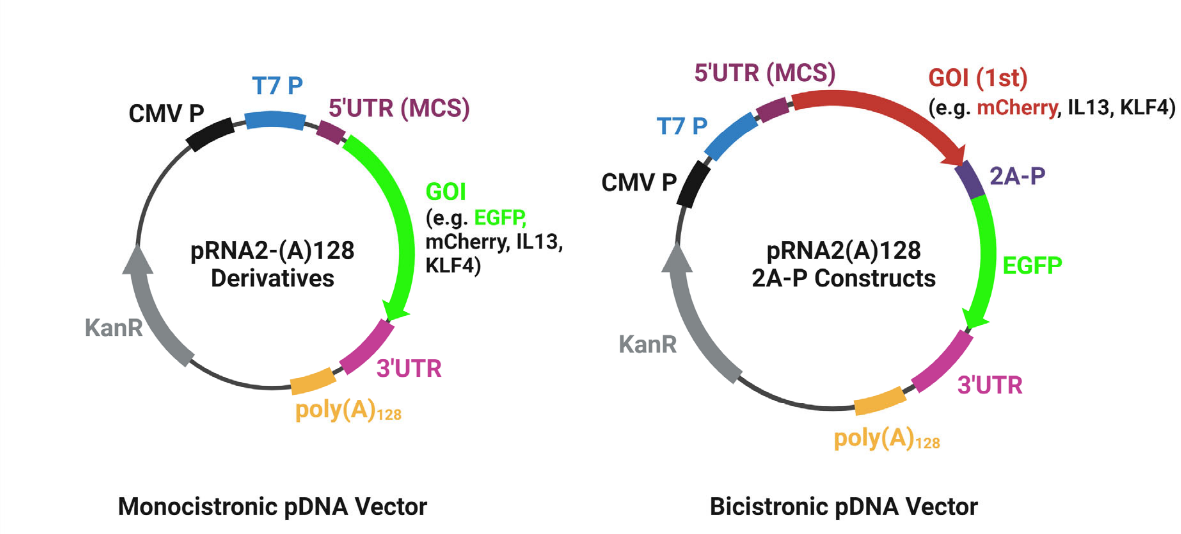

Figure S1. Maps of plasmids used either as template for *in vitro* transcription of mRNA or directly transfected into cells. Both have common features such as a CMV promoter, a T7 promoter, multiple cloning sites (MCS) in the 5’UTR, a head-to-tail duplicated human β-globin 3’-UTR, and a stretch of 128 Adenines, named poly(A)128, to ensure the co-transcriptional addition of a poly(A) tail. Kanamycin resistance was also a common feature in both plasmids used for clonal selection. Monocistronic pDNA vectors encompass one position for the gene of interest, e.g. EGFP, mCherry, KLF4, and IL-13 (left panel), whereas bicistronic pDNA templates consist of two genes in a single open reading frame separated by a 2A peptide sequence (2A-P) [3].

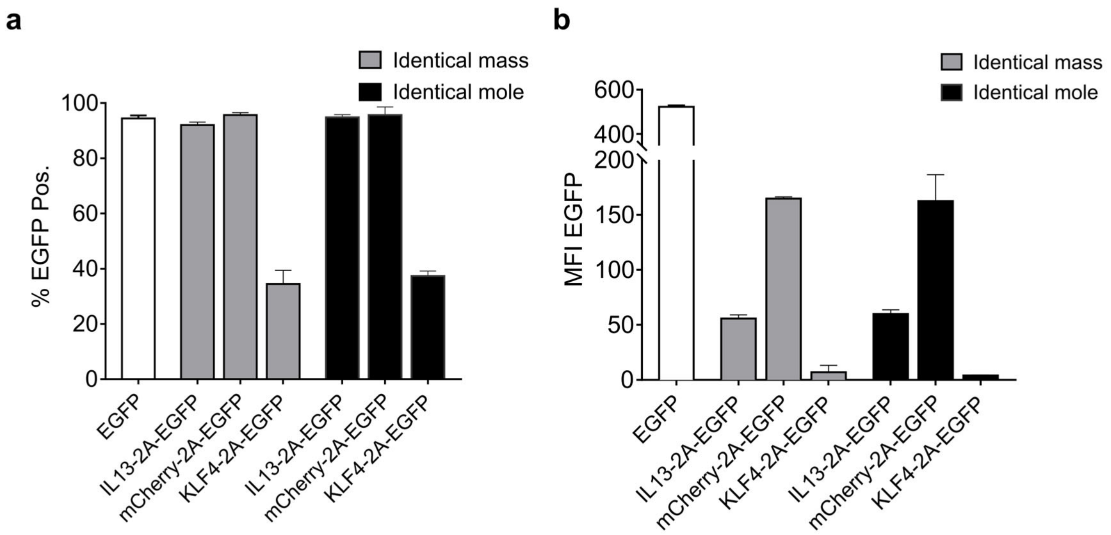

Figures S2. The percentage of EGFP positive population (a) and intensity of EGFP signal (mean fluorescent intensity) (b) reflecting the EGFP expression were quantified using flow cytometric evaluation of cells for transfection of bicistronic 2A constructs, provided in comparison to the results from monocistronic co-transfection according to the figure 2b, and c, respectively.

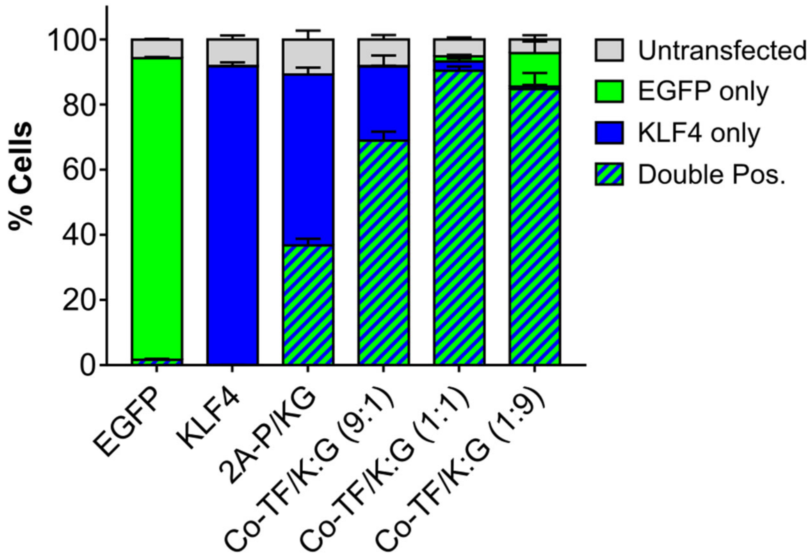

Figure S3. Distribution of transgene expression patterns plotted upon individual transfections, 2A-P, and CoTF of Hela cells with KLF4 and EGFP encoding IVT mRNA by flow cytometric evaluation. Error bars indicate SD for three independently performed experiments.

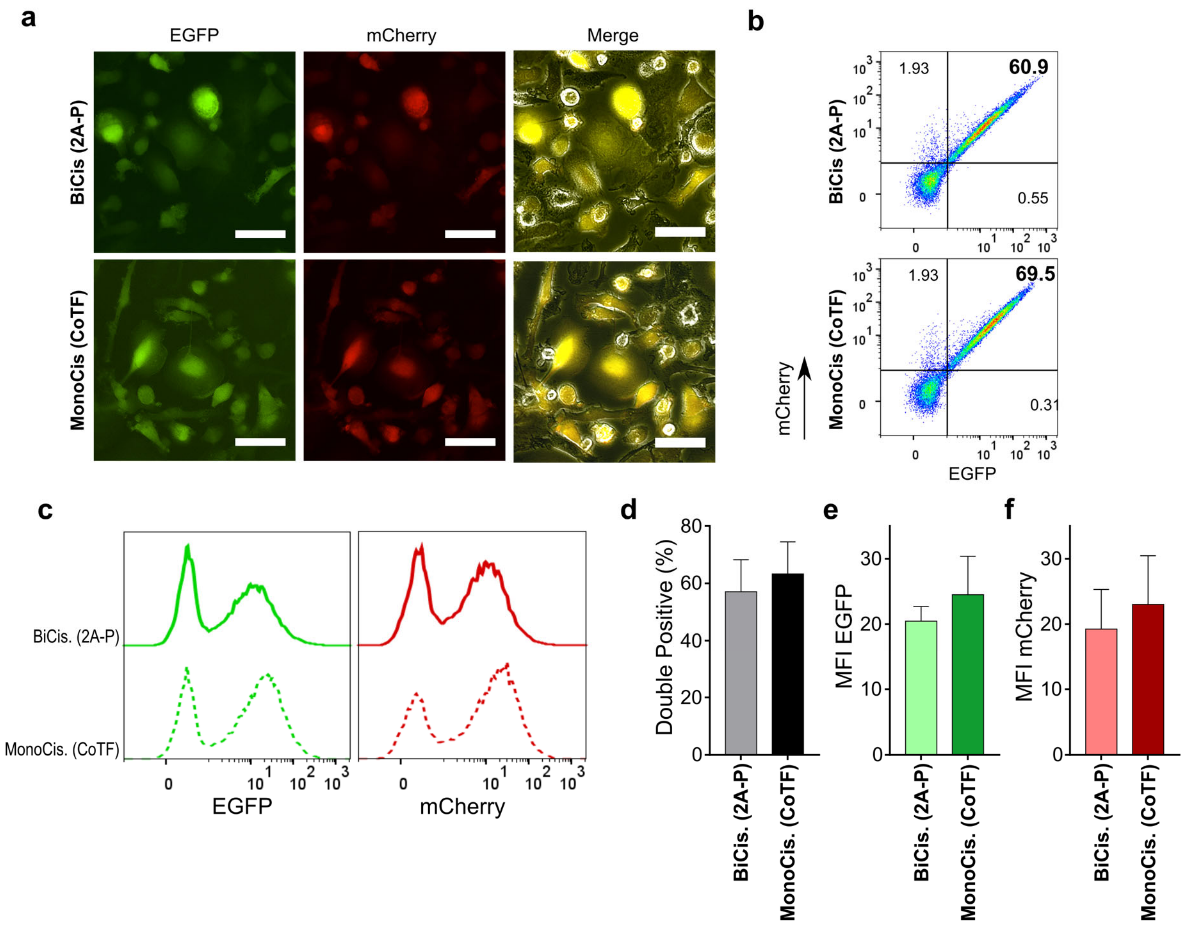

Figure S4. Macrophages transfected with mCherry-2A-EGFP or co-transfected with separate mCherry and EGFP coding IVT-mRNA. (a) Fluorescent images presented in EGFP channel (left panel), mCherry channel (middle panel) as well as merged with phase contrast (right panel). **(b)** Density plots of mCherry-2A-EGFP (upper graph) and mCherry co-transfected with EGFP (lower graph). **(c)** Overlaid histograms of EGFP and mCherry to compare the fluorescence intensity of the transfected cells, solid lines represent the BiCis and dashed lines correlate to MonoCis transfection. **(d)** Percent of double positive cells, **(e)** mean fluorescent intensity (MFI) of EGFP signal and **(f)** mCherry signal are plotted and compared within the two groups; Values are presented as mean ± SD, n≥3, from independent donors each. Numbers indicated within dot plots represent % of cells inside the corresponding gate. Scale bar = 50 µm.

Table S1. The equimass versus equimolar experiment with IVT-mRNA

Payload design	Experiment	Genes	Total mass (ng)	Amont of 1st gene mass (mole)	Amont of 2nd gene (EGFP) mass (mole)
**MonoCis (co-transfection)**	**Equal mass**	IL13+EGFP	500	**250 ng** (0.873 pmol)	**250 ng** (0.612 pmol)
		mCherry + EGFP	500	**250 ng** (0.6525 pmol)	**250 ng** (0.612 pmol)
		KLF4 + EGFP	500	**250 ng** (0.4135 pmol)	**250 ng** (0.612 pmol)
	**Equal mole**	IL13+EGFP	636.7	265 ng (**0.925 pmol**)	371.7 ng (**0.925 pmol**)
		mCherry + EGFP	625.7	305.4 ng (**0.797 pmol**)	320.3 ng (**0.797 pmol**)
		KLF4 + EGFP	586.8	352.5 ng (**0.583 pmol**)	234.3 ng (**0.583 pmol**)
**BiCis (transfection)**	**Identical mole (to standard)**	IL13-2A-EGFP	670.6	1.241 pmol	
		mCherry-2A-EGFP	778.4	1.241 pmol	
		KLF4-2A-EGFP	1064.0	1.241 pmol	
	**Identical mass (to standard)**	IL13-2A-EGFP	500	**0.925 pmol**	
		mCherry-2A-EGFP	500	**0.797 pmol**	
		KLF4-2A-EGFP	500	**0.583 pmol**	

*The standard transfection condition (reference point): **500ng ≈ 1.241pmol** mRNA per well of 12-well plate.

Table S2. The molar amounts of genes, which were used in equimass experiments.

		Gene	Mass amount (ng)	Molecular size (nts/bps)	Molar amount (pmol)
mRNA	MonoCis	IL13 (1st gene)	500	891	1.746
		mCherry (1st gene)	500	1192	1.305
		KLF4 (1st gene)	500	1881	0.827
		EGFP (2nd gene)	500	1250	1.224
	BiCis	IL13-2A-EGFP	1000	1681	1.850
		mCherry-2A-EGFP	1000	1951	1.594
		KLF4-2A-EGFP	1000	2668	1.166
pDNA	MonoCis	IL13 (1st gene)	1000	4821	0.336
		mCherry (1st gene)	1000	5122	0.316
		KLF4 (1st gene)	1000	5811	0.278
		EGFP (2nd gene)	1000	5180	0.312
	BiCis	IL13-2A-EGFP	2000	5611	0.577
		mCherry-2A-EGFP	2000	5881	0.550
		KLF4-2A-EGFP	2000	6598	0.491

Reference

1. H. Moradian, T. Roch, A. Lendlein and M. Gossen: mRNA Transfection-Induced Activation of Primary Human Monocytes and Macrophages: Dependence on Carrier System and Nucleotide Modification. *Sci Rep* **10**, 4181 (2020).
2. H. Moradian, A. Lendlein and M. Gossen: Strategies for simultaneous and successive delivery of RNA. *J Mol Med (Berl)* **98**, 1767 (2020).
3. D.J. Williams, H.L. Puhl and S.R. Ikeda: A Simple, Highly Efficient Method for Heterologous Expression in Mammalian Primary Neurons Using Cationic Lipid-mediated mRNA Transfection. *Front Neurosci* **4**, 181 (2010).
